# Leveraging Color M-Mode to Diagnose Aorto-Atrial Fistula as a Complication of Infective Endocarditis

**DOI:** 10.1155/2024/7550403

**Published:** 2024-05-25

**Authors:** Kramer J. Wahlberg, Matthew A. Kluge, William E. Hopkins

**Affiliations:** ^1^ Department of Medicine Division of Cardiology at the Robert Larner MD College of Medicine University of Vermont, Burlington, VT, USA; ^2^ Department of Medicine The Lifespan Cardiovascular Institute at Rhode Island The Warren Alpert Medical School Brown University, Providence, RI, USA

## Abstract

Aorto-atrial fistula is a rare and life-threatening complication of infective endocarditis, classically diagnosed by visualizing a connection between the aorta and atrium with associated continuous flow. A patient presented with bioprosthetic and native valve enterococcal endocarditis with multiple complications, including an aorto-atrial fistula that was diagnosed by color M-mode on transesophageal echocardiography. We review the features of aorto-atrial fistula and utilize this case to demonstrate how M-mode can be leveraged to provide improved temporal resolution in the setting of diagnostic uncertainty.

## 1. Background

Aorto-atrial fistulas are rare, life-threatening aberrant connections between the aorta and heart chambers [1]. Aorto-atrial fistulas may be congenital and a sequela of ruptured sinus of Valsalva aneurysm, but many are acquired as a consequence of chest trauma, cardiac surgery, aortic dissection, cardiac catheterization, or infective endocarditis, among others [1–3]. Given the inherent variability in predisposing conditions, the clinical presentation can vary depending upon the underlying etiology, anatomy, and shunt physiology of the aorto-atrial fistula which can involve the left or right atrium. Common cardiovascular imaging modalities such as transthoracic echocardiography (TTE) usually lack the sensitivity to visualize complicated anatomy and flow associated with small aorto-atrial fistulae; therefore, a high index of suspicion is warranted to pursue more advanced cardiovascular imaging modalities including transesophageal echocardiography (TEE) and cross-sectional imaging including computed tomography (CT) to diagnose this rare condition that confers a high mortality [4–8] The aim of this study is to review a presentation of aorto-atrial fistula complicating bioprosthetic aortic valve (AV) endocarditis and demonstrate how color M-mode echocardiography was leveraged to establish the diagnosis.

## 2. Case Description

**Figure 1:**
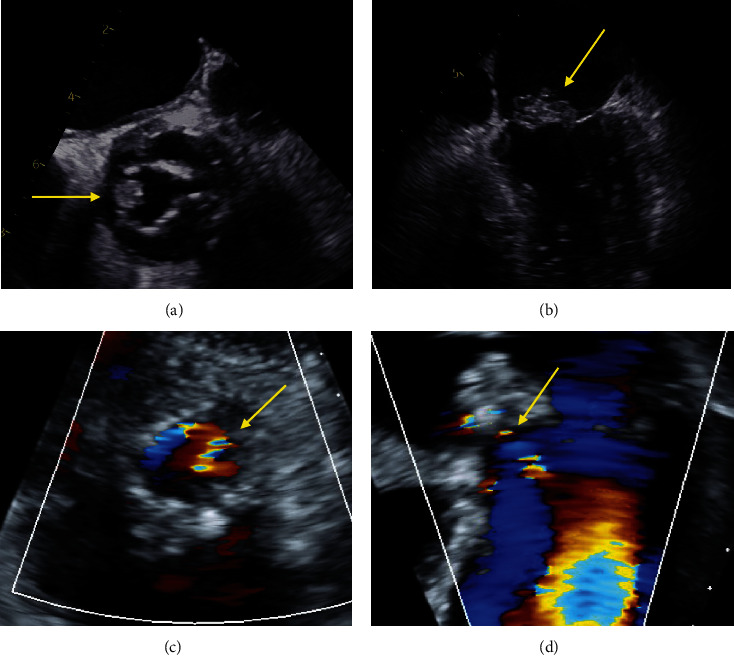
Select images from the (a, b) initial transesophageal echocardiogram performed upon admission to the referring hospital and (c, d) follow-up transthoracic echocardiogram performed upon transfer to our tertiary academic medical center. (a) Midesophageal short-axis view demonstrating thickened bioprosthetic aortic valve leaflets with vegetation (arrow). (b) Midesophageal four-chamber view showing large vegetation along the anterior leaflet of the mitral valve. (c) Parasternal short-axis view of bioprosthetic aortic valve in diastole with color flow suggestive of aortic valve regurgitation (arrow). (d) Apical five-chamber view in diastole showing high-velocity flow in the left ventricular outflow tract consistent with aortic valve regurgitation (arrow).

A very functional 71-year-old male with a history of 25 mm Carpentier-Edwards bioprosthetic AV replacement in 2019 and ascending aorta repair for bicuspid AV stenosis with aortopathy initially presented to a community hospital for evaluation of subacute, progressive confusion and back pain found to have fever, leukocytosis, anemia, and mild troponin-I elevation. A systolic murmur was heard along the right upper sternal border and apex. Blood cultures were drawn given evidence of systemic inflammation and concern for infection. The patient declined admission due to concerns about healthcare costs and returned home. The next day, blood cultures returned positive with high-grade *Enterococcus faecalis* bacteremia, and the patient returned to the hospital for further management of enterococcal bacteremia and evaluation of suspected endocarditis. The source of bacteremia was thought to be possibly related to hemorrhoidectomy 3 months prior to admission with delayed wound healing and limited postoperative care. He was treated with empiric ceftriaxone 2 g every 12 hours and intravenous amoxicillin 2 g every 4 hours. Initial requests to transfer to our tertiary medical center were declined due to our hospital being over capacity. A TTE demonstrated a 2.3 × 1.6 cm mobile vegetation on the anterior leaflet of the mitral valve with trivial regurgitation, a AV bioprosthesis with thickened leaflets and trivial regurgitation, and normal left and right ventricular systolic function. ECG showed sinus rhythm with mild first-degree AV block (PR interval 207 ms). Further evaluation included contrast-enhanced computed tomography (CT) of the abdomen and pelvis with findings suspicious for L1-L2 discitis, and magnetic resonance imaging (MRI) of the head with multiple, bilateral areas of restricted diffusion concerning for cardioembolic strokes. Given evidence of native mitral valve endocarditis and a bioprosthetic AV that was not fully visualized, a TEE was performed that demonstrated the mitral valve vegetation but also visualized a 1.3 × 1.8 cm vegetation involving the AV bioprosthesis (Figures 1(a) and 1(b)) with what was described as a small amount of paravalvular regurgitation without signs of abscess. He was initially stabilized on empiric antibiotic therapy with a course complicated by worsening anemia requiring transfusion suspected to be related to hemolysis.

On hospital day 7, he developed acutely worsening encephalopathy, fevers, and acute hypoxemic respiratory failure secondary to acute decompensated heart failure with pulmonary edema and bilateral pleural effusions prompting transfer to the medical intensive care unit at our tertiary academic medical center. Upon arrival, respiratory status improved with intravenous diuresis, but the patient remained encephalopathic with persistent bacteremia. On the day of admission, a repeat TTE was performed that showed interval elevation in gradients across the AV bioprosthesis (mean gradient 40 mmHg, as compared to 20 mmHg) with mild-moderate regurgitation (Figures 1(c) and 1(d)), a large vegetation along the anterior mitral valve leaflet extending along the aorto-mitral curtain with possible leaflet perforation and at least mild regurgitation, as well as severe left atrial dilation and pulmonary hypertension. Given the diagnosis of *E. faecalis* endocarditis involving AV bioprosthesis and native mitral valve with persistent bacteremia complicated by strokes, discitis, and possible meningitis, surgery was indicated and recommended.

A multidisciplinary approach including critical care, infectious disease, cardiology, cardiothoracic surgery, neurology, and hospital medicine was directed towards the assessment of risk and optimization for possible surgical intervention. While surgery was indicated for infective endocarditis, there was significant concern regarding the risks of exposure to intraoperative systemic anticoagulation in the setting of recent cerebral infarctions suspected to be septic emboli. A rapid and comprehensive, interdisciplinary workup was undertaken that includes MRI of the head and spine. Antibiotic therapy at this time included ceftriaxone 2 grams every 12 hours, intravenous amoxicillin 2 grams every 4 hours, and gentamicin that was added due to persistent bacteremia and concern for possible concurrent neurological infection. Subsequent clinical course notable for paroxysmal atrial fibrillation and occlusion of the left profunda femoral artery suspected to be cardioembolic, lumbar osteomyelitis, and epidural abscess, as well as the discovery of subarachnoid hemorrhage associated with embolic strokes. ECG showed sinus rhythm with prolonged AV delay as compared to prior (221 ms). Urgent cardiothoracic surgery was initially deferred due to the increased risk of worsening intracranial bleeding in the setting of high-dose intraoperative systemic anticoagulation.

**Figure 2:**
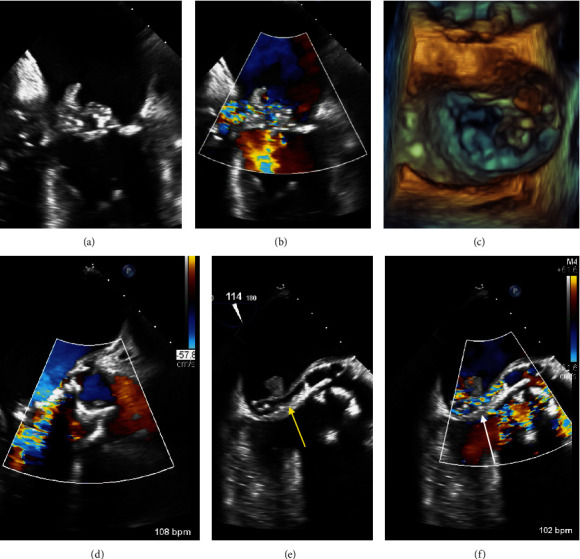
Transesophageal echocardiogram obtained approximately two weeks into hospital stay. (a–c) Bulky vegetation on the mitral valve extending along the aorto-mitral curtain with associated mitral regurgitation and possible leaflet perforation that are not well-characterized. (d) High-velocity flow in the LVOT concerning for regurgitation, as well as (e) an abnormal lucency along the aorto-mitral curtain consistent with abscess and possible fistula (yellow arrow). (f) There was high-velocity flow associated with the abscess by color Doppler (white arrow); however, in the context of tachycardia and adjacent LVOT flow, it was difficult to discern the timing of flow in the cardiac cycle.

**Figure 3:**
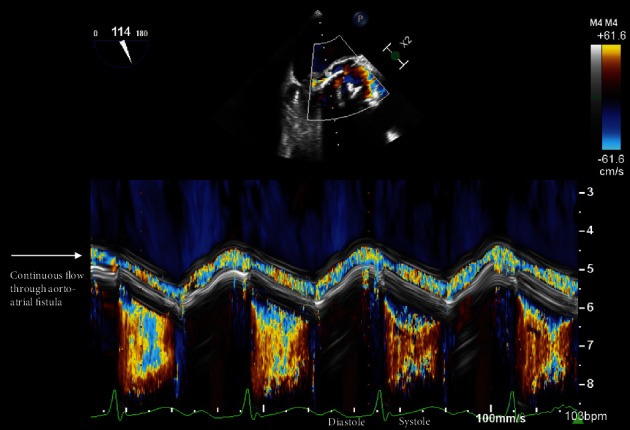
Color M-mode was used to differentiate flow along the aorto-mitral curtain from aortic regurgitation in the LVOT. The temporal resolution from M-mode clearly defines continuous, turbulent flow within the space adjacent to the aorto-mitral curtain, consistent with fistula between the aorta and left atrium. Flow within the LVOT occurs predominantly in systole, which is inconsistent with aortic regurgitation.

Approximately one week after transfer, another TEE was performed to better characterize the AV bioprosthesis and aortic root prior to surgery (Figure 2). The TEE showed interval enlargement of the bulky vegetation along the atrial aspect of the anterior leaflet of the mitral valve extending up to the aorto-mitral curtain associated with anterior leaflet perforation and a significant amount of mitral regurgitation that was difficult to quantify. The AV bioprosthesis appeared to be rocking, the leaflets were significantly thickened with vegetations, and there was edema in the paravalvular region consistent with abscess. Adjacent to the AV was a lucency along the aorto-mitral with associated turbulent flow that was difficult to characterize. There was some degree of AV regurgitation that was technically challenging to interrogate given tachycardia, a large amount of flow in the left ventricular outflow tract (LVOT) in systole, and turbulent flow in the adjacent aorto-mitral curtain suspicious for abscess. Color M-mode was used to provide the temporal resolution necessary to delineate the location and timing of flow with respect to the cardiac cycle (Figure 3). The color M-mode image clearly shows the presence of continuous, turbulent flow throughout systole and diastole within the aorto-mitral curtain, consistent with a fistulous connection thought to be from the aorta to the left atrium. A specific fistula opening in the left atrium was not visualized on two-dimensional (2D) or three-dimensional (3D) echocardiography. Furthermore, there was little flow in the LVOT during diastole suggesting that the lesion previously thought to be aortic regurgitation may have been mischaracterized by TTE.

The patient underwent a high-risk redo sternotomy, and the AV bioprosthesis was found to be encased with vegetations. There was an associated abscess and dehiscence of the intervalvular fibrosa with fistulization from the aorta to the left atrium, confirming the above M-mode findings. Affected areas were excised, and double patch reconstruction of the intervalvular fibrosa was performed using bovine pericardium, accompanied by bioprosthetic aortic and mitral valve replacements, and aortic aneurysm replacement. Operative fluid and tissue cultures yielded no growth. The postoperative course is complicated by mixed vasodilatory, cardiogenic and hemorrhagic shock, acute renal failure requiring hemodialysis, ventilator-associated pneumonia, bilateral lower extremity dry gangrene, heart block requiring permanent pacemaker placement, and persistent hypoxemic respiratory failure ultimately unable to be weaned from mechanical ventilation leading to percutaneous tracheostomy and gastrostomy tube placement. Despite slow but steady improvements in the postoperative period, through discussions mediated by palliative care, it was determined that the impaired functional status and quality of life with possible ventilator dependence, hemodialysis, and possible need for lower extremity amputations were not consistent with the patient's goals of care and the decision was made to transition towards a comfort-directed treatment plan.

## 3. Discussion

Aorto-atrial fistula is a rare complication of infective endocarditis and confers high mortality. Diagnosis can be difficult and typically requires a high index of suspicion to pursue advanced imaging to visualize the anatomy and flow characteristic of an abnormal connection between the aorta and atrium. We describe a case of bioprosthetic AV endocarditis complicated by abscess and aorto-atrial fistula, wherein the diagnosis of aorto-atrial fistula was augmented with color M-mode TEE.

Transthoracic echocardiography is the guideline-recommended first-line test for evaluation of suspected endocarditis; however, the sensitivity of TTE for aorto-cavitary fistula is low [7, 9]. TTE may show continuous high-velocity flow suggestive of a connection between the aorta and low-pressure atrium; however, the spatial resolution of 2D TTE is often inadequate to visualize fistula anatomy and establish the diagnosis [1]. Thus, the diagnosis of aorto-cavitary fistula is typically made via advanced imaging including TEE and CT [5, 10]. Guidelines acknowledge the role of multimodality imaging in the evaluation of infective endocarditis, particularly in a patient with complex anatomy or prosthetic material [8, 11]. TEE is often reported as the test of choice, providing an assessment of anatomy and color flow Doppler necessary to visualize the fistulous connection with associated continuous flow sufficient to diagnose aorto-atrial fistula [4]. CT is less invasive but may be less sensitive and has less special resolution than TEE with regard to visualizing small vegetations or the intricate anatomy associated with aorto-cavitary fistulae [12]. While echocardiography can offer a better assessment of valve morphology and can assess flow with Doppler, in cases of acoustic shadowing from prosthetic valve material, it may not visualize paravalvular or extracardiac complications of endocarditis, such as abscess or pseudoaneurysm as well as CT. Nuclear imaging modalities such as fluoro-18-fluorodeoxyglucose positron emission tomography/computed tomographic angiography or labeled white blood cell scans can be complementary, particularly in the evaluation of prosthetic valve endocarditis [5].

**Figure 4:**
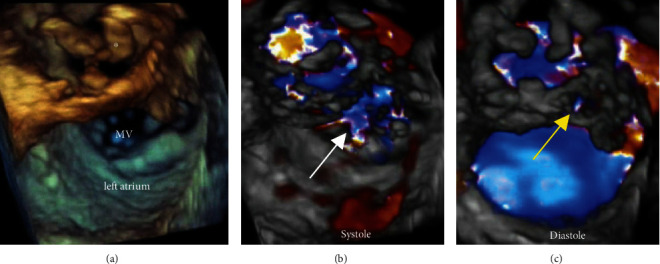
Three-dimensional transesophageal echocardiography. (a) Reference three-dimensional echocardiography showing thickened aortic valve leaflets (∗). (b, c) Three-dimensional color Doppler images suggesting continuous flow across the aorto-mitral continuity in both systole ((b) color denoted by a white arrow is multifactorial from mitral regurgitation and fistula flow) and diastole ((c) yellow arrow) supporting the diagnosis of aorto-atrial fistula.

In this case, TTE demonstrated a large amount of high-velocity flow in the LVOT adjacent to the intervalvular fibrosa. With the acoustic shadowing from the AV prosthesis and aortic repair, it was difficult to visualize the anatomy of the aortic root and adjacent structures. As such, an initial diagnosis of aortic regurgitation secondary to suspected bioprosthetic valve endocarditis was made given the vegetations and dysfunctional prosthetic valve visualized on initial TEE. Only upon repeat TEE as part of the presurgical evaluation was the eventual diagnosis of aorto-atrial fistula established, highlighting how this rare diagnosis requires an ongoing high index of suspicion. Clinically, a complication of endocarditis was suspected given interval clinical deterioration following a period of initial stability with recrudescence of fever, PR interval prolongation, and development of heart failure. During the TEE, we discovered findings concerning for fistula; however, given the prior diagnosis of aortic regurgitation, we utilized color M-mode to provide the temporal resolution necessary to differentiate these two critically different pathologies. By color M-mode, the continuous, turbulent flow in the fistula was clearly visualized throughout systole and diastole, whereas the turbulent flow in the LVOT occurred only in systole, inconsistent with significant aortic regurgitation. Complementary echocardiographic approaches to color M-mode in this situation could include continuous wave Doppler, which could demonstrate continuous flow within the fistula space. Ideally, color 3D echocardiography of the atrium would be performed to identify the atrial orifice of the aorto-atrial fistula, which could be helpful with surgical planning [7, 8]. Indeed, Figure 4 shows representative 3D TEE images of apparent color flow through the aorto-mitral continuity in both systole and diastole supporting the imaging diagnosis of fistula.

With the advent of 2D and 3D echocardiography and improvements in ultrasound technology leading to higher frame rates and resolution, M-mode echocardiography has become increasingly underutilized and underappreciated outside of board exams. Much of this owes to the improved diagnostic accuracy of 2D echocardiography or spectral Doppler in assessing chamber dimensions or valvular pathology in most circumstances. However, at 1000 frames per second or greater, M-mode still offers the best temporal resolution of any echocardiography mode by far. M-mode allows an imager to precisely determine the movement of cardiac structures in relation to the cardiac cycle and should be employed whenever there is doubt. Additional examples where M-mode may have diagnostic utility include mitral valve systolic anterior motion in hypertrophic obstructive cardiomyopathy, mitral valve prolapse, diastolic collapse of the right ventricular free wall in tamponade, and left ventricular septal motion including the “septal bounce” of constrictive pericarditis [13].

## 4. Conclusion

Aorto-atrial fistula is a rare, life-threatening complication of infective endocarditis that requires timely and accurate diagnosis to facilitate appropriate management. This case illustrates the challenges of defining the extent of endocarditis, and how M-mode can be leveraged to provide improved temporal resolution in the setting of diagnostic uncertainty.

## Data Availability

There are no data sets associated with this study. Original images are available upon request.
